# Seasonal Pattern in Gestational Diabetes Mellitus in Poland: A Retrospective Cohort Study

**DOI:** 10.3390/biology12111376

**Published:** 2023-10-27

**Authors:** Marek J. Walkowiak, Małgorzata Jamka, Marcin Piotr Walkowiak, Paweł Gutaj, Ewa Wender-Ożegowska

**Affiliations:** 1 Department of Reproduction, Poznan University of Medical Sciences, Polna Str. 33, 60-535 Poznan, Poland; pawelgutaj@ump.edu.pl; 2 Department of Pediatric Gastroenterology and Metabolic Diseases, Poznan University of Medical Sciences, Szpitalna Str. 27/33, 60-572 Poznan, Poland; mjamka@ump.edu.pl; 3 Department of Preventive Medicine, Poznan University of Medical Sciences, Święcickiego Str. 6, 60-781 Poznan, Poland; marcinwalkowiak@ump.edu.pl

**Keywords:** pregnancy, gestational diabetes, season, glucose tolerance, insolation

## Abstract

**Simple Summary:**

This article examines pregnancy results in a changed metabolism, with more frequent glucose intolerance and subsequent gestational diabetes. Metabolic disturbances may significantly influence foetal development and neonatal health; therefore, a routine checkup of glucose tolerance is performed. Several factors may influence the prevalence of gestational diabetes, including ambient temperature, light exposure, and vitamin D levels. The published data related to seasonal impact are contradictory. We have retrospectively analysed a large cohort of newborns and their mothers. In total, 30,205 newborns and their epidemiological and clinical characteristics were evaluated. We documented that the prevalence of gestational diabetes in Central Europe varied depending on the season; that is, it appears more frequently during the summer, while its prevalence was lowest during the winter (seasons with higher vs. lower insolation). The seasonal variation in the prevalence of gestational diabetes affects both mothers of babies born at term and prematurely.

**Abstract:**

The existing literature does not address the question of the seasonal impact on pregnancy in Central-Eastern Europe; therefore, this study was designed to investigate the seasonal variation in gestational diabetes mellitus (GDM) based on a recent Polish sample. The data of 30,205 newborns from singleton pregnancies and their mothers, including the date and gestational age of birth, neonatal sex and weight, maternal age and parity, mode of delivery, ethnicity, and a detailed list of comorbidities (including GDM), were retrospectively analysed. The prevalence of GDM was significantly (*p* < 0.0001) lower in spring (14.71%) than in the other seasons (16.78%). A higher incidence of GDM was observed for mothers who underwent an oral glucose tolerance test from June to August compared to those who were tested from December to February (17.34% vs. 14.75%, *p* < 0.0001). Similarly, there were significant differences between seasons with higher and lower insolation. The regression analysis revealed that seasonal patterns were significantly associated with the prevalence of GDM. In conclusion, this large retrospective cohort study demonstrated seasonal changes in GDM risk. The observed seasonal patterns may equally refer to mothers of babies born at term and prematurely. Further research concerning GDM risk and other seasonal and gender associations is warranted.

## 1. Introduction

Birth weight is one of the most critical neonatal health indicators, with low and high birth weights associated with subsequent health issues and measurable life outcomes [1]. The suggested aetiology of seasonal birth weight fluctuations is the change in sunlight exposure, which impacts vitamin D production. As a result, this may lead to measurable foetal weight variability. Vitamin D deficiency is associated with the risk of gestational diabetes mellitus (GDM). Not only does this contribute to higher birth weight, but GDM also increases the risk of gestational hypertension and preeclampsia, which lead to low birth weight.

The relationship between vitamin D and GDM is relatively well established. This association is partly explained by higher body mass index (BMI) [2]. Indeed, overweight people tend to have lower 25(OH)D levels, owing to adipose tissue-trapping of the fat-soluble vitamin D [3]. The vitamin D level in the first trimester is considered crucial [4,5], though studies show that the second-trimester level still maintains some relevance [6]. Analyses of the seasonal impact on GDM have revealed mixed results [7]. For example, there was no evidence of a seasonal impact [8], or a very subtle impact [9], in Australia, where the climate is relatively warm and sunny. Previous findings suggest a subtle mechanism that is susceptible to local factors. Since the amount of sunlight varies based on latitude, such seasonal effects may only be present in some regions. Moreover, vitamin D does not have to be photolysed from provitamin D; it can also be sourced from a balanced diet or supplements. Vitamin D deficiency might lead to subclinical conditions with very subtle impacts. Even seasonal infections may be partially mitigated through vaccination or non-pharmaceutical interventions. Those factors are highly local and condition-specific, especially when their net effects on birth weight could be partially nullified.

The existing literature does not address the question of the seasonal impact on pregnancy in Central-Eastern Europe. This region has a humid continental climate [8,10] with a relatively low solar influx, though additional heat is brought by the Gulf Stream. This study was designed to investigate seasonal variation in GDM based on a recent Polish pregnancy sample from 2016 to 2021.

## 2. Materials and Methods

This retrospective cohort study analysed data that were collected from the electronic database of gestations from February 2017 to February 2022 at Poznan University Hospital (a tertiary obstetrical hospital). All neonates were born from singleton pregnancies, and their mothers were included in the analysis. The analysed data included the date and gestational age of birth, neonatal sex and weight, maternal age, BMI and parity, mode of delivery, ethnicity, and a detailed list of comorbidities. Most of the study cohort were Caucasians. Gestational age was verified and confirmed by ultrasound in the first trimester. GDM was diagnosed according to the International Association of Diabetes and Pregnancy Study Groups (IDASPG) criteria [11] and Polish standards [12,13] (Supplementary Tables S1 and S2). The exclusion criteria were type 1 or type 2 pregestational diabetes mellitus. The birthdate was used to determine the season of birth and conception. Specifically, seasons were defined as follows: spring, March–May; summer, June–August; autumn, September–November; and winter, December–February. The insolation at birth and conception was referred to as the average solar radiation incident on a horizontal earth surface for a particular month. This was averaged for that month over 22 years (from July 1983 to June 2005). The insolation threshold (higher vs. lower) was set at 3.0 kWh/m^2^/day according to www.gaisma.com (months >3.0 kWh/m^2^/day: April–August).

Statistical analysis was performed using the Statistica 13.0 software (TIBCO Software Inc., Palo Alto, CA, USA). The level of significance was set at *p* < 0.05, and a confidence interval of 95% was applied. The normality of the data distribution was assessed by the Shapiro–Wilk test, with Levene’s test used to assess the equality of variances. Numerical data were presented as mean and standard deviation (SD) and Boolean as number (percentage (%)). For continuous variables, unpaired *t*-tests were used to compare two groups, with an analysis of variance for months and seasons. Post hoc comparisons were performed with Tukey’s significantly different test. A Chi^2^ test was used for the analysis of categorical variables. Univariate (unadjusted) logistic regression analysis was subsequently performed to assess the relationship between season of birth, sex of infants, gestational age at birth, age of mother, BMI of mother, birth weight of infants, mode of delivery, gestational hypertension, and preeclampsia with the prevalence of GDM. To confirm the relationship between the season of birth and gestational diabetes, parameters that were used in the univariate analysis and those that were related to the season were entered into a multivariate logistic regression analysis and adjusted for birth weight ≥ 90 percentile, caesarean birth, age of mother ≥ 35 years, BMI of mother, gestational hypertension, and preeclampsia. Ethical approval was not required due to the retrospective character of the analysis [14]. However, adequate confirmation was obtained (Poznan University of Medical Sciences Bioethics Committee, Poznan, Poland).

## 3. Results

In total, 30,205 newborns were included in the final analysis, and their epidemiological and clinical characteristics are presented in Table 1. There were slightly more male than female infants in the study population. Most infants were born vaginally (57.10%) and at term (90.67%), with a mean gestational age at birth of 38.5 ± 2.4 weeks and a mean birth weight of 3325 ± 635 g. The mean maternal age was 31.0 ± 4.9 years.

Male infants were younger and had a higher birth weight compared to females. Furthermore, a higher proportion of boys were born prematurely (Supplementary Table S3).

No differences in the sex, gestational age, prevalence of preterm birth, or birth weight of infants born in different seasons were documented (Supplementary Table S4). Table 2 presents the characteristics of the study population depending on the season of conception, birth, and diagnosis of GDM. The prevalence of GDM in women who gave birth between March and June was lower (14.77%) than in women who gave birth in other months (17.01%, *p* < 0.0001). The season of conception was also associated with the prevalence of GDM (Table 3). Conceptions that occurred from January to March were associated with a higher incidence of GDM than conceptions that occurred from July to September (17.32% vs. 14.60%, *p* < 0.0001). A higher incidence of GDM was also observed for mothers who underwent an OGTT between June and August compared to those who underwent the test between December and February (17.34% vs. 14.75%, *p* < 0.0001). The prevalence of GDM in mothers who gave birth in spring (14.71%) was significantly lower (*p* < 0.0001) than in women who gave birth during other seasons of the year (16.78%).

The prevalence of GDM depended on the season that OGTT was performed for mothers of children born at term. This association was not statistically significant in mothers of preterm infants (Supplementary Table S5); however, the odds of having GDM in winter in comparison to summer (season of conception) were not lower in mothers of babies born prematurely (OR = 1.1738; 95% CI: 0.8829–1.5606) compared to those who delivered on time (OR = 1.1360; 95% CI: 1.0385–1.2425). Similarly, the odds of diagnosing GMD in OGTT in summer compared to winter were not lower in mothers of babies born prematurely (OR = 1.2565; 95% CI: 0.9376–1.6837) in comparison to those who delivered on time (OR = 1.2090; 95% CI: 1.1039–1.3241).

Table 4 presents the unadjusted regression results, showing that birth in spring, between March and June (or in months with the highest insolation levels); conception between January and March compared to conception between July and September; OGTT performed between December and February vs. between June and August (or months with higher insolation levels); age of mother ≥ 35 years; BMI of mother; infant birth weight ≥ 90 percentile; delivery mode; gestational hypertension; and preeclampsia were significantly related to GDM prevalence. After covariate adjustment, the season of birth, the season of conception, and the season of OGTT continued to be significantly associated with GDM prevalence (Table 5).

## 4. Discussion

This retrospective study showed that the prevalence of GDM varies depending on the season, with GDM more frequently diagnosed during the summer while its prevalence was lowest during the winter. Similarly, there were significant differences in GDM prevalence between seasons with higher and lower insolation. The seasonal pattern remained significant, even after adjusting for covariates. Additionally, our analysis uncovered other noteworthy findings. For example, we found that caesarean sections were more common in pregnant women who delivered boys in December.

It has been suggested that ambient temperature may influence glucose tolerance [15,16,17]. Akanji et al. documented higher end-test OGTT values, both in obese and lean Caucasians [15] and diabetic and non-diabetic Nigerian subjects [16]. Similarly, Schmidt et al. [17] reported that ambient temperature may affect the OGTT results in pregnant women, with the number of abnormal OGTT results doubling on warmer days. Although Moses and Griffiths [8] did not reveal a significant relationship between temperature and fasting glucose, such an association was documented for 2 h OGTT glucose levels; however, the authors concluded that the observed differences were minor and unlikely to be significant in clinical practice. A recent systematic review on climate factors and GDM risk [18] and a systematic review with meta-analysis on the association between season and GDM [19] summarised the available evidence. Preston et al. [18] concluded that although increased GDM risk may be related to climate factors, further research is needed to evaluate the influence of higher ambient temperature and to define the underlying mechanisms. Khoshhali et al. [19], irrespective of some inconsistencies in the outcome of eleven studies that were included in the meta-analysis, documented the seasonal pattern of GDM prevalence (pooled OR = 1.12; 95% CI: 1.03−1.21); however, both groups suggested the need for further research to elucidate the exact mechanisms of the observed association.

The results may have been influenced by the GDM screening and diagnostic criteria that were used in studies included in both systematic reviews [8,17,20,21,22,23,24,25,26,27,28,29,30,31]. Preston et al. [18] summarised the one- and two-step approaches that were used in the papers mentioned above. In the one-step approach (OGTT, 75 g of glucose), different glucose threshold values were used: from 5.1 mmol/L [11,30] to 7 mmol/L [32] for fasting values and from 7.8 mmol/L [32] to 8.5 mmol/L [9,11,26,30] for 2 h values using World Health Organization [32,33] and IADPSG criteria [11]. For IADPSG criteria [11], the two-step approach GDM diagnostic criteria were even more complex [7,20,32,33,34,35], with the first step based either on random glucose with a cut-off level of 6.5 mmol/L [7,32] or a 50g glucose challenge test with abnormal values from 6.5 mmol/L [7,32] to 7.8 mmol/L [32,34,35]. The second step consisted of the assessment of two (fasting and 2 h values) or four criteria (1 h and 3 h in addition). In the first case, the test lasted for 2 h, and at least 1 abnormal value was considered diagnostic of GDM [7,20,32], whereas 3 h for the second and at least two abnormal values were the basis for the diagnosis [34,35]. The cut-off level of abnormal glucose values in OGTT ranged from 5.3 mmol/L [34] to 6.1 mmol/L [20] for fasting values; from 10.0 mmol/L [34] to 10.5 mmol/L [35] for 1 h results; from 7.8 mmol/L [7,21,32] to 9.2 mmol/L [35] for 2 h values; and from 7.8 mmol/L [34] to 8.0 mmol/L [35] for 3 h glucose concentrations. Interestingly, the subanalysis of the two-step procedure [7,21,23,25,28,29] revealed a significant impact on the season in regard to GMD prevalence, which was higher in summer than in the other seasons (pooled OR = 1.28; 95% CI: 1.18−1.40). In contrast, the one-step procedure [9,22,24,26,30] analysis failed to document any impact (pooled OR = 1.00; 95% CI: 0.93−1.08); however, both subanalyses revealed significant heterogeneity. Perhaps it is worthwhile to consider the geographical locations of the cohorts who were included in the subanalyses that had clear seasonal summer impact (Sweden [28], Greece [25], and Israel [29]), a lack of significant seasonal pattern (Taiwan [22], Canada [24], and the United Kingdom [23]), or even the opposite effect (Australia [9,30]). No seasonal association was documented in two recently published studies for Norwegian [36] and Chinese [22] cohorts. It is worth noting that the present study revealed a seasonal pattern of GMD while using the one-step procedure for diagnosis.

The present study demonstrated the noticeable impact of summer on the prevalence of GDM diagnoses according to international standards used in Poland [11], a country with a continental humid climate and warm summer [10]. The one-step procedure for diagnosing GDM was applied, which is contrary to the results of Khoshhali et al.’s subanalysis [19]. This study has some limitations, including a lack of information on vitamin D levels and dietary regime/supplementation. The clinical significance of some documented statistical differences is minor. Nonetheless, it has some strengths, including a large sample size, high homogeneity, and good characterisation of the study population with clear inclusion and exclusion criteria. Moreover, seasonal variation in GDM prevalence was clearly documented.

## 5. Conclusions

In conclusion, we demonstrated a seasonal pattern of GDM risk in this large retrospective cohort study. The seasonal variation in GDM prevalence may equally refer to mothers of babies born in term and born prematurely. Further research concerning GDM risk and other seasonal and gender associations is warranted.

## Figures and Tables

**Table 1:** Characteristics of the study population.

			Total *n* = 30,205
Sex (*n*, %)	Male		15,669 (51.87%)
	Female		14,535 (48.13%)
	Unknown		1 (0.00%)
Gestational age at birth (weeks) ^1^			38.5 ± 2.4
Preterm birth (*n*, %)	Yes		2817 (9.33%)
	No		27,388 (90.67%)
Neonatal birth weight (g) ^1^			3325 ± 635
Age of mother (years) ^1^			31.0 ± 4.9
BMI of mother (kg/m^2^) ^1^			28.02 ± 4.91
Mode of delivery (*n*, %)	Vaginal birth		15,061 (49.86%)
	Caesarean birth		12,959 (42.90%)
	Vaginal operative birth Vacuum Forceps		2036 (6.74%) 149 (0.50%)
GDM (*n*, %)	Yes		4909 (16.26%)
	No		25,296 (83.74%)
Season of birth (*n*, %)	Winter		6957 (23.03%)
	Spring		7694 (25.47%)
	Summer		7876 (26.07%)
	Autumn		7678 (25.43%)
Season of conception (*n*, %)	Winter		7724 (25.57%)
	Spring		7075 (23.42%)
	Summer		7689 (25.46%)
	Autumn		7717 (25.55%)
Season of GDM diagnosis (*n*, %)	Winter		7479 (24.76%)
	Spring		7836 (25.94%)
	Summer		7761 (25.70%)
	Autumn		7129 (23.60%)
Gestational hypertension (*n*, %)	Yes	Without proteinuria	1682 (1.20%)
		With proteinuria	362 (5.57%)
	No		28,161 (93.23%)
Preeclampsia of mother (*n*, %)	Yes		267 (0.88%)
	No		29,938 (99.12%)

GDM—gestational diabetes mellitus. ^1^ Mean ± SD, BMI—body mass index.

**Table 2:** Prevalence of GDM according to the season of conception and GDM diagnosis.

**Season of Conception**						
		**Winter** ***n* = 7724**	**Spring** ***n* = 7075**	**Summer** ***n* = 7689**	**Autumn** ***n* = 7717**	***p***
GDM (*n*, %)	Yes	1131 (17.23%)	1208 (17.07%)	1188 (15.45%)	1182 (15.32%)	0.0005
	No	6393 (82.77%)	5867 (82.93%)	6501 (84.55%)	6535 (84.68%)	
**Season of GDM Diagnosis**						
		**Winter** ***n* = 7479**	**Spring** ***n* = 7836**	**Summer** ***n* = 7761**	**Autumn** ***n* = 7129**	***p***
GDM (*n*, %)	Yes	1103 (14.75%)	1267 (16.17%)	1346 (17.34%)	1193 (16.73%)	<0.0001
	No	6376 (85.25%)	6569 (83.83%)	6415 (82.66%)	5936 (83.27%)	
**Season of Birth**						
		**Winter** ***n* = 6957**	**Spring** ***n* = 7694**	**Summer** ***n* = 7876**	**Autumn** ***n* = 7678**	***p***
GDM (*n*, %)	Yes	1193 (17.15%)	1132 (14.71%)	1268 (16.10%)	1316 (17.14%)	<0.0001
	No	5764 (82.85%)	6562 (85.29%)	6608 (83.90%)	6362 (82.86%)	

GDM—gestational diabetes mellitus.

**Table 3:** Prevalence of GDM according to the month of conception and GDM diagnosis.

**Month of Conception**														
		**January** ***n =* 2821**	**February** ***n* = 2281**	**March** ***n* = 2375**	**April** ***n* = 2286**	**May** ***n* = 2414**	**June** ***n* = 2528**	**July** ***n* = 2566**	**August** ***n* = 2595**	**September** ***n* = 2406**	**October** ***n* = 2648**	**November** ***n* = 2663**	**December** ***n* = 2622**	***p***
GDM (*n*, %)	Yes	486 (17.23%)	397 (17.40%)	412 (17.35%)	380 (16.62%)	416 (17.23%)	409 (16.18%)	388 (15.12%)	391 (15.07%)	326 (13.55%)	419 (15.82%)	437 (16.41%)	448 (17.09%)	0.0032
	No	2335 (82.77%)	1884 (82.60%)	1963 (82.65%)	1906 (83.38%)	1998 (82.77%)	2119 (83.82%)	2178 (84.88%)	2204 (84.93%)	2080 (86.45%)	2229 (84.18%)	2226 (83.59%)	2174 (82.91%)	
**Month of GDM Diagnosis**														
		**January** ***n* = 2575**	**February** ***n* = 2329**	**March** ***n* = 2574**	**April** ***n* = 2678**	**May** ***n* = 2584**	**June** ***n* = 2781**	**July** ***n* = 2555**	**August** ***n* = 2425**	**September** ***n* = 2239**	**October** ***n* = 2456**	**November** ***n* = 2434**	**December** ***n* = 2575**	*p*
GDM (*n*, %)	Yes	396 (15.38%)	315 (13.53%)	399 (15.50%)	436 (16.28%)	432 (16.72%)	487 (17.51%)	431 (16.87%)	428 (17.65%)	375 (16.75%)	417 (16.98%)	401 (16.47%)	392 (15.22%)	0.0054
	No	2179 (84.62%)	2014 (86.47%)	2175 (84.50%)	2242 (83.72%)	2152 (83.28%)	2294 (82.49%)	2124 (83.13%)	1197 (82.35%)	1164 (83.25%)	2039 (83.02%)	2033 (83.53%)	2183 (84.78%)	
**Month of Birth**														
		**January** ***n* = 2539**	**February** ***n* = 2162**	**March** ***n* = 2644**	**April** ***n* = 2512**	**May** ***n* = 2538**	**June** ***n* = 2539**	**July** ***n* = 2668**	**August** ***n* = 2669**	**September** ***n* = 2663**	**October** ***n* = 2616**	**November** ***n* = 2399**	**December** ***n* = 2256**	***p***
GDM (*n*, %)	Yes	434 (17.09%)	369 (17.07%)	383 (14.49%)	378 (15.05%)	371 (14.62%)	379 (14.93%)	441 (16.53%)	448 (16.79%)	471 (17.69%)	454 (17.35%)	391 (16.30%)	390 (17.29%)	0.0030
	No	2105 (82.91%)	1793 (82.93%)	2261 (85.51%)	2134 (84.95%)	2167 (85.38%)	2160 (85.07%)	2227 (83.47%)	2221 (83.21%)	2192 (82.31%)	2162 (82.65%)	2008 (83.70%)	1866 (82.71%)	

GDM—gestational diabetes mellitus.

**Table 4:** Unadjusted odds ratios (OR) and 95% confidence intervals (CI) for GDM prevalence.

	OR	95% CI	*p*
Season of birth (Spring vs. other seasons)	0.856	0.796–0.920	<0.0001
Time of birth (March–June vs. other)	0.845	0.791–0.903	<0.0001
Insolation for the season of birth (high vs. low)	0.920	0.864–0.979	0.0083
Season of conception (January–March vs. July–September)	1.225	1.122–1.337	<0.0001
Insolation for the season of conception (high vs. low)	0.971	0.912–1.033	0.3497
Season of GDM diagnosis (December–February vs. June–August)	0.824	0.756–0.899	<0.0001
Insolation for the season of GDM diagnosis (high vs. low)	1.101	1.035–1.171	0.0021
Female infant	1.008	0.949–1.072	0.7871
Gestational age at birth ≥38 weeks	0.941	0.868–1.021	0.1426
Age of mother ≥35 years	1.573	1.471–1.682	<0.0001
BMI of mother (kg/m^2^)	1.081	1.074–1.089	<0.0001
Birth weight ≥90 percentile	1.169	1.057–1.294	0.0024
Vaginal birth	0.916	0.862–0.974	0.0051
Cesarean birth	1.106	1.040–1.176	0.0014
Gestational hypertension	1.425	1.266–1.603	<0.0001
Gestational hypertension with proteinuria	1.469	1.144–1.887	0.0026
Preeclampsia	1.807	1.373–2.381	<0.0001

GDM—gestational diabetes mellitus.

**Table 5:** Adjusted * odds ratios (OR) and 95% confidence intervals (CI) for GDM prevalence.

	OR	95% CI	*p*
Season of birth (Spring vs. others)	0.853	0.790–0.921	<0.0001
Season of birth (March–June vs. other)	0.841	0.784–0.902	<0.0001
Insolation for the season of birth (high vs. low)	0.930	0.870–0.994	0.0328
Season of conception (January–March vs. July–September)	1.226	1.117–1.345	<0.0001
Season of GDM diagnosis (December–February vs. June–August)	0.817	0.746–0.896	<0.0001
Insolation for the season of GDM diagnosis (high vs. low)	1.113	1.042–1.188	0.0014

GDM—gestational diabetes mellitus. * Adjusted for birth weight, caesarean birth, age of mother, BMI of mother, gestational hypertension, and preeclampsia.

## Data Availability

The data presented in this study are available upon request from the corresponding authors.

## Supplementary Materials

The following supporting information can be downloaded at: https://www.mdpi.com/article/10.3390/biology12111376/s1, Table S1: Screening algorithm for diagnosis of gestational diabetes mellitus (GDM) or diabetes in pregnancy (DIP); Table S2: Threshold values for diagnosis of gestational diabetes mellitus (GDM) or diabetes in pregnancy (DIP) used for screening; Table S3: Characteristics of the study population according to sex; Table S4: Characteristics of the study population according to the season of birth; Table S5: Prevalence of GDM depending on the term of birth and season of conception or GDM diagnosis. References [11,12,13] are cited in the supplementary materials.
