# Supplementary material for: Seasonal Pattern in Gestational Diabetes Mellitus in Poland: A Retrospective Cohort Study
Source: Biology (Basel). 2023 Oct 27;12(11):1376. doi: 10.3390/biology12111376 (PMC10669897; doi:10.3390/biology12111376)
Supplement: Supplementary file 1 [file biology-12-01376-s001.zip › biology-2642483-supplementary.pdf]

Supplementary Table 1. Screening algorithm for diagnosis of gestational diabetes mellitus (GDM) or overt diabetes in pregnancy (DIP[1]

First prenatal visit		Second trimester (24-26 weeks of gestation)
**All Low-risk women**	**All High-risk women***	**All women who were not previously found to have GDM**
Fasting plasma glucose, OGTT** if FPG*** 93-125 [mg/dl]	OGTT**	OGTT**

*Women having at least one of risk factors[2]: BMI ≥ 30kg/m^2, GDM in previous pregnancy, Type 2 Diabetes in 1st degree female relatives, Delivering a baby with weight ≥ 4500g in previous pregnancy
**75-g Oral Glucose Tolerance Test
*** Fasting plasma glucose

Supplementary Table 2. Threshold values for diagnosis of gestational diabetes mellitus (GDM) or overt diabetes in pregnancy (DIP) used for screening[3]

	**GDM**	
**Glucose measure**	[mg/dl]	[mmol\l]
**Fasting plasma glucose**	92-125	5.1-6.9
**OGTT* 1-h plasma glucose**	>180	>10
**OGTT* 2-h plasma glucose**	153-199	8.5-11
	**Overt Diabetes in Pregnancy (DIP)**	
**Glucose measure**	[mg/dl]	[mmol\l]
**Fasting plasma glucose**	≥126	≥7
**OGTT* 2-h plasma glucose**	≥200	≥11.1
**Random plasma glucose**	≥200	≥11.1

*75-g Oral Glucose Tolerance Test

1. REGULATION ORDINANCE OF MINISTER OF HEALTH of August 16, 2018. On the organizational standard of perinatal care [in Polish] https://www.gov.pl/web/zdrowie/zmiany-w-organizacji-opieki-okoloporodowej-1-stycznia-2019-r-wchodzi-w-zycie-nowy-standard-organizacyjny available online: https://www.gov.pl/attachment/31ff3347-061c-413f-a843-5b6f574b9928 acessed on 1 September 2023
2. Wender-Ożegowska, E.; Bomba-Opoń, D.; Brązert, J.; Celewicz, Z.; Czajkowski, K.; Gutaj, P.; Malinowska-Polubiec, A.; Zawiejska, A.; Wielgoś, M. „The Polish Society of Gynaecologists and Obstetricians standards for the management of patients with diabetes" [in Polish]. *Ginekol Perinatol Prakt* **2017**, *2*, 215–229. https://journals.viamedica.pl/ginekologia_perinatologia_prakt/article/view/56571
3. International Association of Diabetes and Pregnancy Study Groups Consensus Panel; Metzger, B.E.; Gabbe, S.G.; Persson, B.; Buchanan, T.A.; Catalano, P.A.; Damm, P.; Dyer, A.R.; Leiva, A. de; Hod, M.; Kitzmiler, J.L.; Lowe, L.P.; McIntyre, H.D.; Oats, J.J.N.; Omori, Y.; Schmidt, M.I. International Association of Diabetes and Pregnancy Study Groups Recommendations on the Diagnosis and Classification of Hyperglycemia in Pregnancy. *Diabetes Care* **2010**, *33*, 676–682. https://doi.org/10.2337/dc09-1848.

Supplementary Table 3. Characteristics of the study population according to sex.

		Male<br>*n* = 15,669	Female<br>*n* = 14,535	*p*
**Gestational age at birth (weeks)**[1]		38.4 ± 2.4	38.5 ± 2.3	<0.0001
**Preterm birth (*n*, %)**	**Yes**	1541 (9.83%)	1275 (8.77%)	0.0015
	**No**	14,128 (90.17%)	13,260 (91.23%)	
**Birth weight (g)**[1]		3395 ± 642	3250 ± 617	<0.0001
**Age of mother (years)**[1]		31.0 ± 4.9	31.0 ± 4.9	0.6812
**Mode of delivery (*n*, %)**	**Vaginal birth**	7544 (48.15%)	7516 (51.71%)	<0.0001
	**Caesarean birth**	6844 (43.68%)	6115 (42.07%)	
	**Vaginal operative birth**			
	**-Vacuum**	1180 (7.53%)	856 (5.89%)	
	**-Forceps**	101 (0.64%)	48 (0.33%)	
**GDM (*n*, %)**	**Yes**	2538 (16.20%)	2371 (16.31%)	0.7871
	**No**	13,131 (83.80%)	12,154 (83.69%)	
**Season of birth (*n*, %)**	**Winter**	3544 (22.62%)	3413 (23.48%)	0.1720
	**Spring**	3991 (25.47%)	3702 (25.47%)	
	**Summer**	4081 (26.05%)	3795 (26.11%)	
	**Autumn**	4053 (25.86%)	3625 (24.94%)	
**Season of conception (*n*, %)**	**Winter**	4072 (25.99%)	3652 (25.13%)	0.2481
	**Spring**	3628 (23.15%)	3447 (23.72%)	
	**Summer**	3950 (25.21%)	3739 (25.72%)	
	**Autumn**	4019 (25.65%)	3697 (25.44%)	
**Season of GDM diagnosis (*n*, %)**	**Winter**	3861 (24.64%)	3618 (24.89%)	0.2050
	**Spring**	4083 (26.06%)	3752 (25.81%)	
	**Summer**	4089 (26.10%)	3672 (25.27%)	
	**Autumn**	3636 (23.20%)	3493 (24.03%)	

GDM – gestational diabetes mellitus

[1] Mean±SD

Supplementary Table 4. Characteristics of the study population according to the season of birth.

		Winter *n* = 6957	Spring *n* = 7694	Summer *n* = 7876	Autumn *n* = 7678	*p*
**Sex (*n*, %)**	**Male**	3544 (50.94%)	3991 (51.87%)	4081 (51.82%)	4053 (52.79%)	0.2437
	**Female**	3413 (49.06%)	3702 (48.12%)	37795 (48.18%)	3625 (47.21%)	
**Gestational age at birth (weeks)**[1]		38.5 ± 2.3	38.5 ± 2.4	38.5 ± 2.3	38.5 ± 2.4	0.2725
**Preterm birth (*n*, %)**	**Yes**	660 (9.49%)	733 (9.53%)	750 (9.52%)	674 (8.78%)	0.3002
	**No**	6297 (90.51%)	6961 (90.47%)	7126 (90.48%)	7004 (91.22%)	
**Birth weight (g)**[1]		3321± 636	3316 ± 648	3336 ± 629	3327 ± 626	0.2246

[1] Mean±SD

Supplementary Table 5. Prevalence of GDM depending on the term of birth and season of conception or GDM diagnosis

		Preterm birth *n* = 2817					Birth on time *n* = 27,388				
		**Season of conception**									
		**Winter *n* = 708**	**Spring *n* = 690**	**Summer *n* = 697**	**Autumn *n* = 722**	***p***	**Winter *n* = 7016**	**Spring *n* = 6385**	**Summer *n* = 6992**	**Autumn *n* = 6995**	***p***
**GDM (*n*, %)**	**Yes**	122 (17.23%)	108 (15.65%)	105 (15.06%)	105 (14.54%)	0.5330	1209 (17.23%)	1100 (17.23%)	1083 (15.49%)	1077 (15.40%)	0.0011
	**No**	586 (82.77%)	582 (84.35%)	592 (84.94%)	617 (85.46%)		5807 (82.77%)	5285 (82.77%)	5909 (84.51%)	5918 (84.60%)	
		**Season of GDM diagnosis**									
		**Winter *n* = 663**	**Spring *n* = 749**	**Summer *n* = 723**	**Autumn *n* = 682**	***p***	**Winter *n* = 6816**	**Spring *n* = 7087**	**Summer *n* = 7038**	**Autumn *n* = 6447**	***p***
**GDM (*n*, %)**	**Yes**	93 (14.03%)	112 (14.95%)	123 (17.01%)	112 (16.42%)	0.4032	1010 (14.82%)	1155 (16.30%)	1223 (17.38%)	1081 (16.77%)	0.0004
	**No**	570 (85.97%)	637 (85.05%)	600 (82.99%)	570 (83.58%)		5806 (85.18%)	5932 (83.70%)	5815 (82.62%)	5366 (83.23%)	

GDM – gestational diabetes mellitus
